# Molecular identification of *Pentatrichomonas hominis* in animals in central and western Thailand

**DOI:** 10.1186/s12917-021-02904-y

**Published:** 2021-06-02

**Authors:** Aongart Mahittikorn, Ruenruetai Udonsom, Khuanchai Koompapong, Rachatawan Chiabchalard, Chantira Sutthikornchai, Preeyaporn Monatrakul Sreepian, Hirotake Mori, Supaluk Popruk

**Affiliations:** 1 Department of Protozoology, Faculty of Tropical Medicine, Mahidol University, Ratchawithi Road, Ratchathewi, Bangkok, 10400 Thailand; 2 Faculty of Medical Technology, Rangsit University, Pathum Thani, 1200 Thailand; 3 Department of General Medicine, Faculty of Medicine, Juntendo University, Tokyo, Japan

**Keywords:** *Pentatrichomonas hominis*, Cats, Cattle, Dogs, Thailand

## Abstract

**Background:**

*Pentatrichomonas hominis* inhabits the digestive tracts of several vertebrates, such as humans, monkeys, pigs, dogs, cats and rats. This protozoan was originally considered a commensal of the digestive tract but has subsequently been identified as a potential zoonotic parasite and a causative agent of diarrhoea. Molecular techniques are considered more sensitive and specific to detect *P. hominis.* This study aimed to determine the presence and genetic diversity of *P. hominis* in animals in Thailand. A total of 403 faecal samples were collected from 119 cats, 55 dogs, 73 goats, 35 monkeys, 55 cattle and 66 pigs, and the presence of *P. hominis* was determined using the nested polymerase chain reaction method. Sequence analysis of small-subunit ribosomal RNA genes was used to determine the genotype of the organism.

**Results:**

Twenty-six samples (26/403, 6.45%) were positive for *P. hominis*. The highest prevalence was found in cats (21/119; 17.65%), followed by cattle (3/55; 5.45%) and dogs (2/55; 3.64%). Seven out of 26 nucleotides demonstrated 100% sequence identity with existing sequences; additionally, 16 novel sequence patterns were identified. All nucleotide sequences of *P. hominis*-positive samples were shown in the same branch with the previously described *P. hominis* sequences found in humans, dogs and goat.

**Conclusion:**

This is the first study on *P. hominis* infections in animals in Thailand. Our findings revealed that the prevalence of *P. hominis* was significantly higher in cats than in cattle and dogs. Cats were the main reservoir host; however, *P. hominis* can infect several kinds of animals. Therefore, the proper waste management of animals is necessary to reduce and prevent infection in the community.

## Background

*Pentatrichomonas hominis*, formerly known as *Trichomonas hominis*, is a flagellated protozoan that inhabits the intestinal tracts of humans and animals and was originally believed to be a commensal protozoan [1]. However, studies have indicated *P. hominis* as the causative agent of diarrhoea in mammals [2–5] and gastrointestinal or pulmonary diseases in children and older people [6, 7]. Therefore, the pathogenic potential of this protozoan cannot be ruled out. Infection with this organism is prevalent in dogs, cattle, pigs and monkeys in economically developing regions and industrialised countries [8]. Later studies detected the presence of *P. hominis* in goat [9], water buffalo [5] and farmed wildlife [3]. Little is known about the transmission routes, biology, life cycle, primary host and animal reservoirs of this protozoan [10]. Traditionally, diagnostic methods for the detection of *P. hominis* have relied on microscopic examinations of the stool, which should be conducted immediately; alternatively, the stool material should be immediately preserved in a suitable fixative to preserve the morphological characteristics of the protozoan. However, trophozoites of *P. hominis* can be difficult to differentiate from *Tritrichomonas foetus* because of similarities in their motility and form [11]. Therefore, several polymerase chain reaction (PCR) assays that are considered more sensitive and specific and are now recognised as definitive for the detection of *P. hominis* have been described in the literature [3, 8, 12, 13]*.* The development of molecular detection tools and the increase in the awareness of the zoonotic potential and adaptation of this parasite to a new host have resulted in an increasing number of studies on *P. hominis* [3, 5, 8, 9, 14–16]. Although the potential significance of *P. hominis* as a mammalian pathogen has been recognised, epidemiological studies in humans and animals in Thailand and many other countries are currently lacking. To the best of our knowledge, no studies have been conducted on the prevalence of *P. hominis* in animals in Thailand so far. Detailed investigations including systematic surveys of trichomonads in humans and animals are required to improve our knowledge of the zoonotic origins of trichomonads. This study aimed to determine the prevalence and molecular characterisation of *P. hominis* in dogs, cats, goats, cattle, pigs and monkeys in Thailand to understand the risks and dynamics of infections in humans and animals.

## Results

The overall prevalence of *P. hominis* in animals was 6.45% (26/403). The highest prevalence was observed in cats (21/119; 17.65%), followed by cattle (3/55; 5.45%) and dogs (2/55; 3.64%). Abandoned cats in temples located in the Nakhon Nayok Province were found to be most infected. No *P. hominis-*positive samples were obtained from goats, monkeys and pigs in this study. Table 1 enlists the global prevalence, case reports, diagnostic method and geographical region of *P. hominis* infection in animals.

**Table 1:** Prevalence of *Pentatrichomonas hominis* infection in animals according to the country of identification and the diagnostic method used per published records

Country	Type of animal/source of sample	Detection method	Positive samples/n (%)	Case report	References
Thailand	Cats from a temple in Nakhon Nayok Province	Nested PCR, sequencing	16/79 (20.25)	–	This study
	Cats from a refuge in Kanchanaburi Province		5/40 (12.5)	–	This study
	Dogs from a refuge		2/55 (3.64)	–	This study
	Goats from farms		0/73 (0)	–	This study
	Monkeys in a town		0/35 (0)	–	This study
	Cattle from farms		3/55 (5.45)	–	This study
	Pigs from farms		0/66 (0)	–	This study
Austria	Necropsy, biopsy or organ samples from cats with diarrhoea	In situ hybridisation	1/102 (0.98)	–	[17]
Brazil	Cats with or without diarrhoea	Faecal culture; PCR	3/77 (3.89)	–	[18]
	Cats with chronic diarrhoea	PCR, sequencing	–	2	[11]
China	Dogs from pet hospitals	Microscopy Single-tube nested PCR, sequencing	62/315 (19.7) 99/315 (31.4)	–	[19]
	Police dogs	Nested PCR, sequencing	69/252 (27.38)	–	[8]
	Puppy with diarrhoea	PCRs, sequencing	–	1	[2]
	Goats from farms	Single-tube nested PCR, sequencing	2/781 (0.3)	–	[9]
	Monkeys from a wildlife park	Nested PCR, sequencing	28/60 (46.67)	–	[8]
	Yellow cattle	Nested PCR, sequencing	15/323 (4.6)	–	[5]
	Dairy cattle		36/526 (6.8)	–	[5]
	Water buffalo		1/106 (0.9)	–	[5]
	Pigs from farms	Nested PCR, sequencing	38/158 (24.05)	–	[16]
	Pigs from farms	Nested PCR, sequencing	39/500 (7.8)	–	[20]
	A pig with diarrhoea	PCRs, sequencing	–	1	[21]
	Sheep from farms	Single-tube nested PCR, sequencing	0/832 (0)	–	[9]
	Minks from farms	Nested PCR, sequencing	29/60 (48.33)	–	[3]
	Sika deer from farms		26/130 (20)	–	[3]
	Rex rabbits from farms		13/80 (16.25)	–	[3]
	Blue foxes from farms		27/60 (45)	–	[3]
	Silver foxes from farms		26/60 (43.33)	–	[3]
	Raccoon dogs from farms		32/60 (53.33)	–	[3]
France	Puppies from breeding kennels	PCR, sequencing	34/215 (15.8)	–	[22]
Japan	Cats from public animal shelters	Microscopy	0/1079 (0)	–	[23]
	Kittens in pet shops	Nested PCR, sequencing	2/409 (0.5)	–	[24]
	Puppies in pet shops		38/544 (7)		[24]
	Dogs from public animal shelters	Microscopy	1/906 (0.11)	–	[23]
	Marmosets	PCR, sequencing	58/88 (66)	–	[25]
Korea	Puppy with diarrhoea	PCR, sequencing	–	3	[26]
Poland	Dogs from kennels	Real-time PCR	5/41 (12.19)	–	[27]
United States	Cats attending an international cat show	PCR, sequencing	2/103 (1.9%)	–	[12]
	Kittens with diarrhoea	Microscopy, histology	–	4	[28]
	Dogs with diarrhoea	PCR, sequencing	13/14 (92.85)	–	[4]
	Dogs from a laboratory animal resources facility	PCR, sequencing	0/19 (0)	–	[29]
	Dog faeces submitted to a veterinary diagnostic laboratory for parasitologic analysis		0/81 (0)	–	[29]
	Dogs with diarrhoea		–	4	[29]
	Preputial washing or scraping from bulls	PCR, sequencing	–	4	[15]

n, number

High similarities (≥98%) between the 26 nucleotide sequences of the partial small-subunit ribosomal RNA (SSU rRNA) gene of *P. hominis* and the sequences deposited in GenBank were observed in the present study. Seven out of 26 nucleotide sequences showed 100% identity to the existing sequences (MF991102 [6 out of 7] and MK177545 [1 out of 7]), all of which were from cats. Nineteen out of 26 nucleotide sequences presented with 16 novel sequence patterns (Table 2).

**Table 2:** Accession numbers of the representative positive samples used for phylogenetic reconstruction in this study

Type of animal	No.	GenBank accession No. (type of patterns)	Sequence similarity (%)	Similar GenBank reference sequence
Cats	9	MW074255 (CTM5)	99.30	MK177545
	24	MW074256 (CTM1)	99.65	MF991102
	25	MW074257 (CTM6)	99.65	MK177545
	73	MW074258 (CTM9)	99.62	MH997493
	11	MW074259 (CTM1)	99.65	MF991102
	56	MW074260 (CTM6)	99.65	MK177545
	64	MW074261 (CTM2)	99.65	MF991102
	67	MW074262 (CTM10)	99.25	MH997493
	70	MW074263 (CTM3)	98.95	MF991102
	71	MW074264 (CTM7)	98.90	MK177545
	75	MW074265 (CTM1)	99.65	MF991102
	76	MW074266 (CTM4)	99.30	MF991102
	79	MW074267 (CTM11)	99.23	KX136884
	80	MW074268 (CTM8)	99.28	MK177545
Dogs	15	MW074269 (DTM1)	97.80	MF991102
	38	MW074270 (DTM2)	98.94	MF991102
Cattle	35	MW074271 (CTTM1)	99.52	MF991102
	63	MW074272 (CTTM2)	99.31	MF991102
	75	MW074273 (CTTM3)	98.29	MK177545

**Fig. 1:**
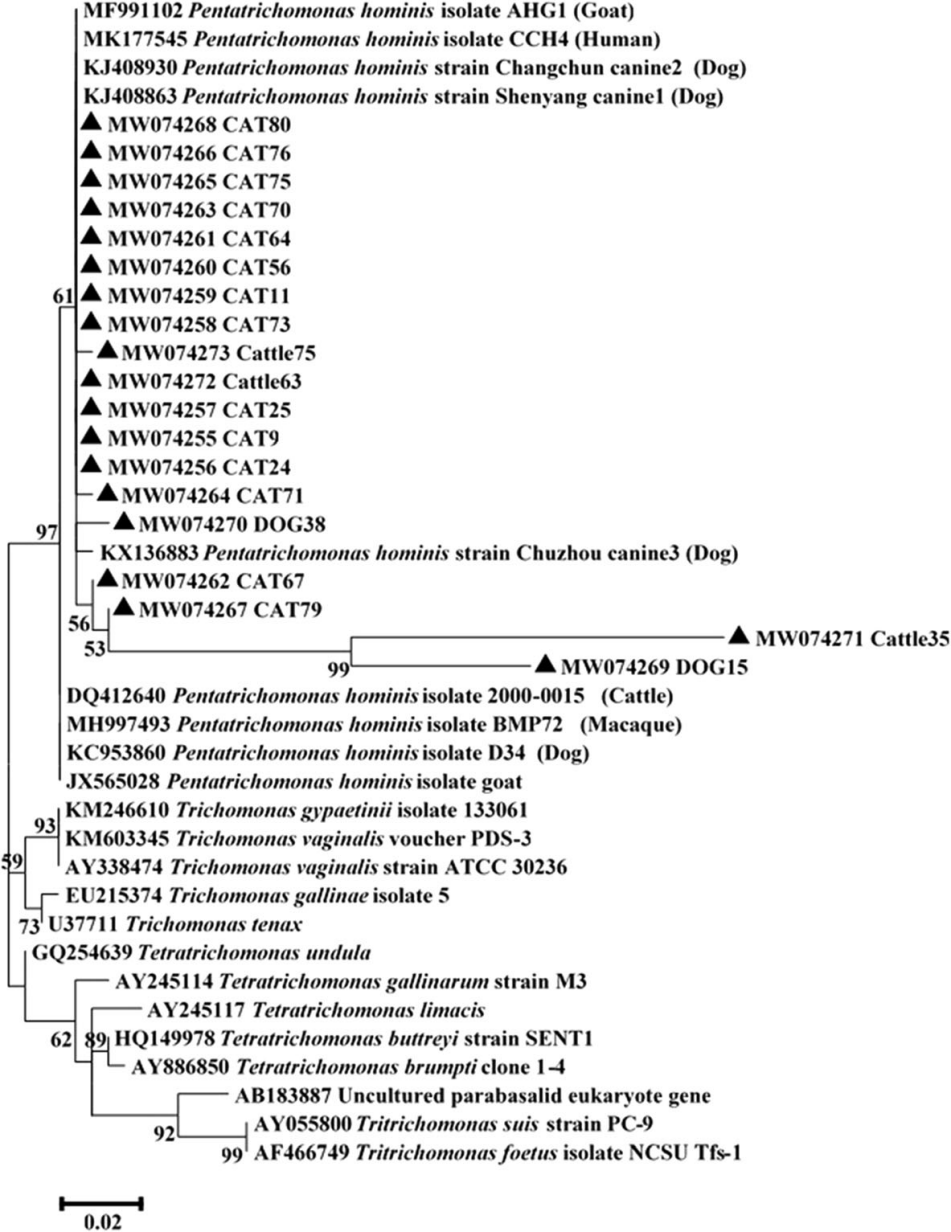
Phylogenetic tree of *P. hominis* isolates and reference sequence of small-subunit ribosomal RNA (SSU rRNA) genes from GenBank (243 positions in the final dataset). Values on nodes represent bootstrap support from the maximum likelihood methods. ▲ Novel types identified in this study

We conducted a phylogenetic analysis of 19 nucleotide sequences from the *P. hominis*-positive samples and compared them with the reference sequences in the GenBank database, as shown in Fig. 1. All nucleotide sequences in the *P. hominis*-positive samples were located within the same branch as that reported in humans, dogs and goats (reference studies).

## Discussion

Several clinical and epidemiological studies employ molecular methods for detecting *P. hominis* from faecal samples [7, 8, 13, 27, 30]. In the present study, nested PCR was used to identify *P. hominis* infections in animals in Thailand. Accordingly, the positive samples were sequenced to identify *P. hominis* SSU rRNA genes in a convenience population of faecal samples from cats, dogs, goats, pigs, cattle and monkeys. A high prevalence of *P. hominis* infections has been identified in these animals previously [10]. To the best of our knowledge, this is the first report of *P. hominis* infections in animals in Thailand.

The overall prevalence of *P. hominis* in animals in this study was 6.45%. The prevalence of *P. hominis* depends on geographical areas, animal species and diagnostic methods. Clearly, the molecular method most widely used in the literature is specific and more sensitive in detecting *P. hominis* than any other method because neither requires viable trophozoites nor an expert microscopist. Given a similar method for detection, the prevalence varies among countries (Table 1). One of the main findings of this study was the high prevalence of this protozoan in cats (17.65%)—which was higher than those reported in the USA, Japan and Brazil [12, 18, 23, 24]—followed by cattle (5.45%) and dogs (3.64%). This may be due to the high density of cats within limited spaces (temples and refuges), which increases the chances of infection from faecal contamination. Additionally, the natural behaviour of grooming among cats supports the transmission of infection through physical contamination [11, 24]. The prevalence of *P. hominis* in dogs in this study was lower than those reported in China, Japan, South Korea and Poland [8, 24, 26, 27]. However, similar to previous reports, *P. hominis* infections were dominant in cats and dogs in the present study [19, 22, 24, 31]. Younger age and abnormal (liquid or semiliquid) faeces have been linked to an increased risk of *P. hominis* infection in dogs [19, 22]. However, previous studies have reported that *P. hominis* has the potential for diarrhoea in dogs, cats and humans [1, 11, 26]. Clinical cases of *P. hominis* infection with chronic diarrhoea have been observed in many animals (Table 1). Unfortunately, although the present study did not record the characteristics of animal faeces, doing so would explain the association between *P. hominis* infection and faeces type better. Moreover, the cats, dogs and monkeys did not have real owners; thus, their precise ages remained unknown.

The transmission of *P. hominis* was believed to occur directly between hosts, likely through the faecal–oral route via the ingestion of trophozoites [11]. However, recent studies have shown that *P. hominis* can form a pseudocyst stage under unfavourable environmental conditions, thus allowing the parasite to survive for several days outside the environment of the host [2, 5, 7, 22]. Consequently, the possibility of transmission via a pseudocyst cannot be ruled out. Further molecular epidemiological investigations including the age of the animals and the characteristics of the faeces are required to determine the risk of *P. hominis* to humans.

The prevalence of *P. hominis* in cattle in the present study was consistent with that reported by Li et al. (2020) [5]. As was observed in dogs [19, 22], the prevalence of *P. hominis* was significantly higher in cattle with abnormal faeces but was not different between pre-weaned calves, post-weaned calves, juveniles and adult cattle [5]. Based on our molecular detection and sequencing results, the several isolates obtained from cats, dogs and cattle were confirmed to be *P. hominis-*positive. These findings implied that cats, dogs and cattle could act as natural hosts of *P. hominis*, which are consistent with the results of previous studies [2, 5, 24, 27]. *P. hominis* may be a potential organism for zoonotic transmission in people who are in close contact with infected animals or consume water contaminated with *P. hominis* [5, 7]. According to Kamaruddin et al. (2014) [30], close contact with animals may be the potential risk factor for *P. hominis* human infection.

Although recent reports have documented that *P. hominis* infection can occur in goats [9, 14, 30], pigs [20] and monkeys [8], no positive sample was obtained in the present study. Therefore, the risk of zoonotic transmission of *P. hominis* from goats, pigs and monkeys in central Thailand is considered minimal. Given the settings observed in the goat and pig farms, where the animals are in close proximity to each other, the spread of an infection, if present, would be fast. Intestinal trichomonads, including *P. hominis*, are shed into the environment at the trophozoite stage and can survive for several days in the faeces leading to environmental contamination [7, 22, 32].

Previous studies showed a high prevalence of *P. hominis* in pigs and monkeys in China [8, 16, 20]. However, the prevalence of *P. hominis* in goat was much lower in China, the Philippines and Indonesia [9, 14, 30]. These differences might be attributed to differences in the age of the animals, the immunity when the stool samples were collected, the geographical location and the detection methods used. In this study, two previously described (CCH4 [human] and AHG1 [goat]) and 16 novel types of *P. hominis* were detected in the animal samples. All known genotypes were found in the cat samples, implying that they were not host-specific. Phylogenetic analyses revealed that the 16 novel genotypes were clustered in the same branch with the human, goat and dog samples.

In Thailand, apart from the data obtained from this study, little is known about the prevalence and genotype of *P. hominis* in humans and animals. Thus, additional epidemiological and genotyping studies of *P. hominis* are warranted.

## Conclusion

The present study is the first to report the prevalence of *P. hominis* in animals in central and western Thailand by employing a molecular technique. *P. hominis* was highly prevalent in cats, followed by cattle and dogs indicating that cats may be the main natural host of *P. hominis*. Sixteen novel and two known genotypes were found in the animal samples, indicating that *P. hominis* may not be host-specific. We recommend the proper waste management of animals in the community, particularly in temples, refuges and farms to control *P. hominis* infections in restricted areas. As the first study to report the molecular epidemiological data on *P. hominis* infection among animals in Thailand, our study has some limitations. Firstly, given that it is a cross-sectional study, the findings can only be related to a certain time period. Secondly, samples were collected from different types of animals and different locations, and the sample size and species types were limited. Therefore, further studies with larger sample sizes (in each animal species), more animal species and wider survey sites are required to investigate the zoonotic potential of *P. hominis*.

## Methods

### Sample collection

A total of 403 stool samples were collected from 119 abandoned cats (79 from a temple in the Nakhon Nayok Province) (Central Thailand) and 40 samples from a refuge in the Kanchanaburi Province (Western Thailand), 55 abandoned dogs (from a refuge in the Nakhon Nayok Province), 73 goats (from farms in the Ayutthaya Province in Central Thailand), 35 monkeys (from a town in the Lopburi Province in Phra Prang San Yod), 55 cattle (from farms in the Ayutthaya Province) and 66 pigs (from farms in the Ayutthaya Province) between 2016 and 2020. The abandoned cats and dogs were fed by the Buddhist monks in a temple and caretakers in refuges.

The age of the cattle was > 6 months, whereas those of the goats and pigs were 4–12 and 2–8 months, respectively. The exact ages of the cats, dogs and monkeys were not known because they did not have real owners. All the animals were healthy and allowed to roam freely, except for the cats, goats and pigs, which were kept in big cages or pens. The stool samples were collected from the grounds immediately after defecation, stored in cool conditions during transportation and preserved at − 20 °C for DNA extraction.

The study protocol was approved by the Ethics Committee at the Faculty of Tropical Medicine-Animal Care and Use Committee, Mahidol University (FTM-ACUC 017/2020E).

### PCR amplification

A fragment of the partial SSU rRNA genes from the extracted DNA was amplified using nested PCR. The PCR products were approximately 339 base pairs (bps) in length. The outer primer set was Ph1 (5′-ATGGCGAGTGGTGGAATA-3′) and Ph2 (5′-CCCAACTACGCTAAGGATT-3′) [8]. The inner primer set was Th3 (5′-TGTAAACGATGCCGACAGAG-3′) and Th5 (5′-CAACACTGAAGCCAATGCGAGC-3′) [13]. Each 25 μL reaction mixture contained 1 × PCR buffer, 1.5 mM MgCl_2_, 0.2 mM dNTPs, 1 μM of each primer and 2.5 U of *Taq* DNA polymerase (Fermentas, USA). The PCR cycling conditions of the primary round were as follows: *initial* denaturation at 94 °C for 5 min, 35 cycles of denaturation at 95 °C for 60 s, annealing at 59 °C for 60 s and extension at 72 °C for 60 s, followed by a final extension at 72 °C for 7 min. The PCR cycling conditions for the second round were the same as those for the primary rounds, except for the annealing temperature (61 °C). The PCR products from the second round were 339 bps; they were separated on 2% agarose gel and visualised under a UV transilluminator.

### Sequencing and phylogenetic analysis

All positive PCR products of the 339 bp fragment from the *P. hominis* SSU rRNA gene obtained from the secondary PCR reaction were purified and sequenced in both directions using an ABI 3730xl automated DNA analyser (Basic Canada Inc., Ontario, Canada). The nucleotide sequences from all positive samples were tested by comparing the homology with those of the *P. hominis* sequences reported in the GenBank database, using a BLAST search of the National Center for Biotechnology Information database (https:/blast.ncbi.nlm.nih.gov/Blast.cgi). The representative nucleotide sequences of this study were deposited in GenBank under the following accession number MW074255-MW074273.

The nucleotide sequences of the *P. hominis*-positive samples and 25 reference sequences were manually edited using the BioEdit v.7.2.5 Software (Ibis Biosciences, Carlsbad, CA, USA), and multiple alignments were performed using ClustalW (Table 1). Finally, a phylogenetic analysis was conducted using the MEGA version 6 software (http://www.megasoftware.net). Evolution of the DNA sequences was best elucidated by the Jukes–Cantor model + gamma distribution. A phylogenetic tree was constructed using the maximum likelihood method and tested with 1000 bootstrap replicates.

### Statistical analysis

Descriptive analyses (percentages) were used to describe the prevalence of *P. hominis* in the stool samples throughout the study regions.

## Data Availability

The datasets used and/or analysed during the present study are available from the corresponding author by reasonable request.

## Abbreviations

bps: base pairs
PCR: Polymerase chain reaction
SSU rRNA: Small-subunit ribosomal RNA
